# Quality of paediatric blood transfusions in two district hospitals in Tanzania: a cross-sectional hospital based study

**DOI:** 10.1186/1471-2431-9-51

**Published:** 2009-08-14

**Authors:** Dominic Mosha, Anja Poulsen, Hugh Reyburn, Elimsaada Kituma, Frank Mtei, Ib C Bygbjerg

**Affiliations:** 1 Kilimanjaro Christian Medical Centre (KCMC), PO Box 3010, Moshi, Tanzania; 2 Joint Malaria Programme, PO Box 2228, Moshi, Tanzania; 3 Department of International Health, Immunology & Microbiology, University of Copenhagen, 5 Ǿster Farimagsgade Building 16, P.O. Box 2099, DK-1014 Copenhagen K, Denmark; 4 Department of Infectious and Tropical Diseases, London School of Hygiene & Tropical Medicine, Keppel Street, London WC1E 7HT, UK

## Abstract

**Background:**

Blood transfusion (BT) can be lifesaving for children; however, monitoring the quality of BT is important. The current study describes the quality of paediatric BT delivered in two district hospitals in north-east Tanzania in order to identify areas for quality assurance and improvement in the administration of BT.

**Methods:**

All 166 children admitted in the paediatric wards and receiving BT through April to June 2007 were prospectively observed. Medical records, request forms and registers in the laboratories were reviewed to identify blood source, blood screening and indications for BT. BT was observation before, during and after transfusion process.

**Results:**

Malaria related anaemia accounted for 98% of the BTs. Ninety-two percent of the children were assessed for paleness. Clinical signs such as difficult breathing and symptoms of cardiac failure were only assessed in 67% and 15% of the children respectively, prior to the BT decision. Pre-transfusion haemoglobin and body temperature were recorded in 2/3 of the patients, but respiratory rate and pulse rate were not routinely recorded. In 40% of BTs, the transfusion time exceeded the recommended 4 hours.

The zonal blood bank (ZBB) and local donors accounted for 10% and 90% of the blood, respectively. ABO and RhD typing and screening for HIV and syphilis were undertaken in all transfused blood. Evidence for hepatitis B or C infection was not checked except in the ZBB.

**Conclusion:**

Criteria for BT are not always fulfilled; time to initiate and complete the transfusion is often unacceptable long and monitoring of vital signs during BT is poor. Blood from the ZBB was often not available and BT often depended on local donors which implied lack of screening for hepatitis B and C. It is recommended that an external supervision system be established to monitor and evaluate the quality of BT performance in the laboratories as well as in wards.

## Background

Blood transfusion (BT) is one of life-saving treatments available in paediatric wards in African hospitals but its use is associated with a number of risks including transfusion reactions, heart failure and transmission of infectious agents [1,2]. For example a Kenyan study found that up to 2% of transfused blood was HIV-infected due to laboratory errors [3].

In spite of these risks many children are transfused without meeting standard criteria for transfusion, the most important of which are severe anaemia with respiratory distress or impaired consciousness (symptomatic severe anaemia), conditions associated with mortality between 20–35% [4].

In children, it is important to minimise delay in transfusion as the majority of deaths occur before BT is started [5]. If there is a delay of BT for more than 24 hours then there is probably no additional benefit to transfusion. During the transfusion children need to be closely monitored for evidence of respiratory distress or transfusions reactions [6].

A large variation in mortality has been reported among children transfused in Kenyan district hospitals [7]. It is likely that this is the result of variations in standards of care in selecting children for transfusion, screening blood or administering BT in a timely and safe way. However very little detail is known about how children are selected for transfusion and how these children are subsequently managed in these settings. We conducted this study over a 3 month period to assess how children were transfused and whether this was in line with current guidelines in 2 hospitals serving populations exposed to high malaria transmission in North-East (NE) Tanzania.

## Methods

### Study hospitals

The study was conducted in two district hospitals each serving predominantly rural populations in a malaria-endemic area of NE Tanzania. Hospital A had considerably more human and technical resources available than hospital B, due to an ongoing research programme (hospital A had 10 doctors and 18 nurses working in the paediatric ward while hospital B had 3 doctors and 9 nurses in its paediatric ward). In 2005 Hospital A and B had 27 and 28 paediatric beds respectively, and 4,266 and 4,447 paediatric admissions. None of the hospital had a fridge for storing blood in the ward and therefore, locally donated blood was not stored.

At the time of the study, nationally recommended indications for transfusion were primarily Hb < 4 g/dl or < 6 g/dl with respiratory distress. Blood screening recommendations included ABO and Rh matching and screening for HIV, syphilis and hepatitis B and C virus [8].

Each hospital had a laboratory to screen and cross match blood for transfusion. In both hospitals a Hemocue (201+, Hemocue AB, Ängelholm Sweden) was used to measure haemoglobin level.

### Data collection

Data were collected during the rainy season between mid April to the end of June, 2007. All children who were transfused in the paediatric ward during the study period were included.

Quality indicators of blood transfusion practices were selected from WHO guidelines [9] and information gathering was as follows; Children who were prescribed for BT were followed from the start by a research assistant who was always available (24 hours a day). A questionnaire recorded the time of decision for blood transfusion (from the case notes or inquiry with the admitting physician) and time of starting and stopping the transfusion. Details of frequency of observations were recorded in most cases by direct observation supplemented by a review of case notes. Laboratory data were collected from routine registers kept in each laboratory supplemented by direct observation.

For quality control of the transfused blood basing on HIV status, two millilitres of blood was taken from the remains in the blood bag after transfusion. Blood was centrifuged and transported to a research laboratory where samples were anonymously tested for HIV using Western Blot^® ^test [10].

### Data Analysis

SPSS version 12 was used for data analysis. Before data analysis, data were double entered in MS Access and data cleaning was done to ensure maximum accuracy and consistence. Numerical variables were summarised into median and range. Categorical variables were summarised using cross tabulation to estimate different proportions, X^2 ^(and chi-trend) and their respective p-values at 5% (2-tail) were also estimated. However, the statistical test to assess for interaction between hospitals was not included.

### Ethics

The study was approved by the Ethical Review Committee of Kilimanjaro Christian Medical Centre. It was authorised by the hospital directors of the two study hospitals and the manager of NE Zone Blood Bank (ZBB) which is the only blood bank serving health facilities within four regions of NE Tanzania.

## Results

In the study period the two hospitals had a total of 1037 paediatric admissions, of these 166 (16%) of the children had a BT (24 of the children at hospital A and the rest at hospital B). Hundred and one (61%) of the recipients were boys and 65 (39%) were girls. The ratio between boys and girls was the same for both hospitals. Twenty-five (15%) of the children had previously received a BT, the majority within the last couple of months.

The median age at time of BT was 1.8 (range 0.1 – 5.4) years for both girls and boys. In hospital A the age of study participants was significantly lower than in Hospital B (median 1.5 against 2.1 years, p < 0.01). Malaria associated with anaemia accounted for 157 (98%) of the BTs, and only 2% had a diagnosis of marasmus, pneumonia or burn.

The clinical signs were recorded before making decisions for BT as shown in Table 1. About 57 (35%) of patients from hospital B were not measured for haemoglobin (Hb) before transfusion. However, this was contrary to hospital A where Hb from all patients was measured before transfusion.

**Table 1:** Clinical signs recorded before blood transfusion

**Clinical sign**	**Present** **n (%)**	**Not present** **n (%)**	**Not described whether present or not** **n (%)**
Cardiac failure/symptom	13 (8)	12 (7)	141 (85)
Hepatomegaly	7 (4)	146 (88)	13 (8)
Respiratory distress	17 (10)	94 (57)	55 (33)
Impaired consciousness	59 (36)	19 (11)	87 (53)
Capillary refill	3 (2)	17 (10)	146 (88)
Dehydration	16 (10)	141 (85)	9 (5)
Pallor	110/36 (66/22)*	7 (4)	13 (8)

*The numerator represents severe pallor and the denominator represents some pallor

There was a wide variation in Hb recorded values prior to BT between patients (ranged from 2 g/dl to 8 g/dl). We also observed the difference in mean Hb before transfusion in the two hospitals. Hospital A have had lower mean Hb (3.9 g/dl) compared to hospital B (4.7 g/dl). This difference was statistically significant (p < 0.05). Less than a quarter, 25 (23%) of the children had Hb < 4 g/dl and 65 (59%) had the Hb between 4 g/dl and 5.9 g/dl. Surprisingly, about 20 (18%) of the children from hospital B had the Hb ≥ 6 g/dl.

Body temperature was recorded in 112 (68%) of the children before, in one child (1%) during and in 23 (14%) after transfusion. Respiratory rate was counted in 42 (25%) before, 3 (2%) during and in 1 (1%) after transfusion. Blood pressure and pulse rate was only measured in a total of 4 and 6 children, respectively.

In both hospitals there was a wide range in time between doctor's decision for a patient to be transfused and the initiation of transfusion (median 2.7 hour and range 35 minutes – 21 hours). The transfusion time (the time between starting and finishing blood transfusion) ranged widely from 2 – 16 hours. In 66 (40%) of the studied patients, the transfusion time was more than 4 hours. Four children had unspecific reactions reported during blood transfusion, and BT was in all cases stopped for a period and then started again. No evidence for a child death due to blood reaction or lack of BT.

The mean dose of the amount of blood prescribed to a patient per kilogramme in the two hospitals was 19.3 (median 20 mls/kg and range 2 – 35 mls/kg). Only 17 (10%) of the children were supplied with blood from the ZBB and 134 (81%) received blood which was donated directly either from relatives or family friends. For the remaining 15 (9%) of the patients there was no information on the source of blood.

A total of 13 (8.2%) deaths among the transfused children occurred on the admission date (all in hospital B) and five (3%) children were referred to the regional hospital. Females were three times more likely to die compared to their males counterpart (Old Ratio = 3.4, 95% CI: 0.9–15.8). However, this difference was not statistically significant. Our findings also suggested that, there was no gender difference in Hb levels pre-transfusion (p-value = 0.467).

The ABO Rhesus grouping and compatibility tests were also reported and documented meticulously in the two hospital laboratories. Donated blood was screened for HIV using a Capillus^® ^test and confirmed by Determine^® ^test. The blood was also screened for syphilis. Surprisingly, none of the studied hospitals performed ELISA or PCR for HIV screening. Additionally, hepatitis B and C virus were only screened for ZBB blood. It was evident from this study that transfused blood was not screened for malaria. We also observed that packed red blood cells were not prepared; instead all children in need of blood were transfused whole blood. In addition transfused children were also given a dose of furosemide to avoid body fluid overload.

A sub-sample of 77 blood samples (29 samples from hospital A and 48 samples from hospital B) from the used blood bag were re-tested for HIV using Western Blood^® ^test. Fortunately, all blood samples tested negative.

## Discussion

This study differs from most other existing studies in developing countries [3,7,11] by focusing on the entire process of BT. Our results demonstrate that it is important to improve the quality of blood transfusion. We have no reason to believe that standards in the two selected hospitals were significantly different from others in the region, and in fact one of the hospitals (hospital A) has considerably more human and technical resources available within the paediatric ward than other hospitals in the area, due to ongoing research activities.

The decision for a BT was either taken by clinical judgment or by measuring the Hb. In a study from Malawi [12] it was shown that clinical features might provide a reasonable basis for indicating the need for BT in areas with poor laboratory setup. However, in our study the clinical assessment was not optimal, one third of the children were not assessed for respiratory distress and 85% had no information whether cardiac failure was present.

Over a third of transfused children did not have Hb measured before BT at hospital B, and among patients in whom pre-transfusion haemoglobin was actually checked 20 (18%) had pre-transfusion haemoglobin level above 6 g/dl and no additional indications to justify transfusion. These findings suggest that clinical judgement can exacerbate blood supply shortage. The results are similar to those of Lackritz *et al *from Kenya [6] where it was judged that half of the paediatric transfusions could have been avoided. Therefore, more has to be done in the current HIV era where there has been focus on ways to avoid HIV transmission.

Fourteen (8%) children had to wait for more than 6 hours from the doctor's decision for BT to the actual initiation of it. The main underlying reason was limited availability of blood. The later has also been reported by the Global Database on Blood Safety [2] and Tanzania National Blood Transfusion Service [13] that shortage of blood supply is a growing problem, particularly in developing countries. The delay might increase the risk of dying due to severe anaemia in children, as most deaths occur within the first hours after admission [14].

The procedure during the BT was far from optimal; vital signs (Respiratory rate, Pulse rate or Blood pressure) were rarely monitored in spite of well recognised hazards of BT [1]. Poor monitoring of these important vital signs, at least in hospital B, might be explained by shortage of staff, however the situation was the same in hospital A with considerably more staff. Thus, lack of training, guidelines and or supervision might be the contributing factors.

To our knowledge the actual time for BTs has not been observed in other studies in Africa. However, long transfusion time increases risk of haemolysis of the transfused blood as well as delaying the time before a viable Hb level is restored [9].

As the study was an observational study, the clinicians and nurses were informed on the aim of the study. Thus, there might have been a bias and the staff might in fact have performed better than normal. The investigators intervened and informed the doctor and or nurse on duty when care was perceived to put a child at risk e.g. long waiting time for a patient to be transfused, prolonged blood transfusion and worsening of the child's clinical condition during blood transfusion. Therefore, the observed deficiencies of performance must be considered a minimum.

Malaria was the leading cause of severe anaemia as found in other studies in the sub-Saharan Africa [15-17], and the mortality of 8.5% is similar to what previously has been reported in Kenya [7]. Fifteen percent of the children in our study have had another BT within the following couple of months, this emphasizes that children in need of BT are a vulnerable group, and there is a need to follow them after discharge.

It was satisfying that all donated blood was tested for HIV, although only Capillus^® ^test (a rapid HIV test) was used and routinely performed in the two hospital laboratories. Using the more sensitive Western Blot^® ^test (a confirmatory) on the selected blood samples, no HIV antibody positive sample was found. However, WHO proposes an ELISA HIV test [18] to be performed on donated blood. The limitation for both Capillus^® ^test and the Western Blot^® ^test is that they identify HIV antibodies and not HIV antigens; in a high HIV prevalence area, the sero-negative window during the HIV incubation period may be important. Hepatitis B and C virus tests were not performed in the two hospitals contrary to National Blood Transfusion Guideline [13].

Our study found that relatives and friends remain a significant source of blood for transfusion in the two study areas compared to ZBB blood. However, it is impressive that the ZBB in only 2 years period in place and its service has reached most hospitals within four regions in north-eastern Tanzania. A Chinese study [19] reported that it is possible to build up a well functioning blood bank system within a few years. Though the conditions in Asia are different from Africa the goal might be to use their system in an adjusted version to establish blood centres in all regions of the country.

## Conclusion

This study confirms many of the findings from other studies in Africa [3,11,17] however, it adds new knowledge on the actual performance of the procedures during the process of BT. These suggest the need for improvements in both laboratory procedures and clinical care. In this part of Tanzania, many of the laboratory problems may be reduced as the Zonal Transfusion service extends its activities.

Our study suggests that it is necessary to improve the quality of BT at all steps within the procedure. Monitoring and evaluation system has to be in place in the hospital settings. One way forward could be that the Ministry of Health develops a standardised form that has to be filled for each BT, and the ZBB bank could be strengthened and play an important role in the quality control (Table 2).

**Table 2:** Problems identified and suggestions on how to solve them

**Problem identified**	**Strategies to improve**	**Responsible authority**
Unnecessary BT	Training/supervision -Use of standardised form-	*MOH/ZBB
BTs often a clinical decision	Ensure fast haemoglobin measurement is possible -Use of standardised form-	Management team within hospital MOH/ZBB
BTs poorly monitored and waiting time is too long	Training/supervision -Use of standardised form-	MOH/ZBB
Recurrent BT in children, the majority within the last couple of months	Training/supervision Routinely outpatient follow-up after BT	Management teams within hospitals
The screening is not optimal	Enhance the capacity of ZBB to be the reliable blood source	ZBB
Majority of donors are still relatives and friends	Enhance the capacity of ZBB to be the reliable blood source	ZBB

*MOH = Ministry of Health

ZBB = Zonal Blood Bank

## Competing interests

The authors declare that they have no competing interests.

## Authors' contributions

All authors participated in design, implementation, analysis and interpretation of the study findings. DM and AP designed the study. DM was the Principle Investigator and responsible for data collection and coordinating study procedures. AP, HR and ICB supervised the progression of the study. DM, AP, HR and ICB were involved in all phase of the study and have full access to all study data. EK was responsible for the laboratory work by handling blood samples and performing confirmatory HIV test. Data summarisation and cleaning was done by FM. Analysis of data and drafting of the manuscript was led by AP and DM. HR and ICB critically reviewed the manuscript and all authors approved the final manuscript.

## Pre-publication history

The pre-publication history for this paper can be accessed here:


